# Tolerance limit of external beam radiotherapy combined with low-dose rate brachytherapy in normal rabbit tissue

**DOI:** 10.1093/jrr/rrad036

**Published:** 2023-05-20

**Authors:** Hang Ling, Wenxiao Huang, Waisheng Zhong, Pingqing Tan, Hailin Zhang, Yong Liu, Jie Chen

**Affiliations:** Department of Otolaryngology Head and Neck Surgery, Xiangya Hospital, Central South University, Xiangya Road, Kaifu District, Changsha, Hunan, 410008, China; Department of Head and Neck Surgery, The Affiliated Cancer Hospital of Xiangya School of Medicine, Central South University, Tongzipo Road, Yuelu District, Changsha, Hunan, 410000, China; Department of Head and Neck Surgery, The Affiliated Cancer Hospital of Xiangya School of Medicine, Central South University, Tongzipo Road, Yuelu District, Changsha, Hunan, 410000, China; Department of Head and Neck Surgery, The Affiliated Cancer Hospital of Xiangya School of Medicine, Central South University, Tongzipo Road, Yuelu District, Changsha, Hunan, 410000, China; Department of Head and Neck Surgery, The Affiliated Cancer Hospital of Xiangya School of Medicine, Central South University, Tongzipo Road, Yuelu District, Changsha, Hunan, 410000, China; Department of Otolaryngology Head and Neck Surgery, Xiangya Hospital, Central South University, Xiangya Road, Kaifu District, Changsha, Hunan, 410008, China; Department of Head and Neck Surgery, The Affiliated Cancer Hospital of Xiangya School of Medicine, Central South University, Tongzipo Road, Yuelu District, Changsha, Hunan, 410000, China

**Keywords:** head and neck, external beam radiotherapy, low-dose rate brachytherapy, rabbit, multimodal therapies

## Abstract

**Background:**

Dosage-optimized multimodal radiotherapies that are safe for head and neck cancer patients are desirable. In this study, we investigated tissue tolerance to varying doses of external beam radiotherapy (EBRT) combined with low-dose rate brachytherapy in the neck of a rabbit model.

**Methods:**

Twenty rabbits were used in the four test groups (five each) with iodine-125 seeds implanted in the neck treated with EBRT in four doses at 50, 40, 30 and 20 Gy each. Twelve rabbits for three control groups (four each). Three months after implantation, all rabbits were euthanized, and target tissues were collected. Analyses included seed implantation assessment, histopathological evaluation, immunohistochemistry staining, terminal deoxynucleotidyl transferase dUTP nick end labeling assay, electron microscopy and statistics with the SPSS software.

**Results:**

Five rabbits died in the four test groups, and three rabbits died in the three control groups (one per group), which showed no significant difference by survival analysis. The calculated minimum peripheral dose was 17.6 Gy, the maximum dose near the seed was 1812.5 Gy, the D90 was 34.5 Gy and the mean dose was 124.5 Gy. In all groups that received radiation, apoptosis occurred primarily in the esophageal mucosa and corresponded to the dose of radiation; a higher dose caused a greater apoptosis, with significant difference between groups (*P* < 0.05). Electron microscopy of carotid arteries revealed that endothelial cells were swollen and some were shed from basement membrane, but no other noticeable tissue damages.

**Conclusions:**

Limited EBRT at maximal dose (50 Gy) combined with the brachytherapy interstitially applied to the neck was tolerated well in the rabbit model.

## BACKGROUND

Interstitial iodine-125 (^125^I) seed implantation as a low-dose rate (LDR) permanent brachytherapy modality has been applied to treat various malignancies, such as prostate cancer, intraocular tumors, intracranial tumors, breast cancer, soft tissue sarcomas and head and neck cancer (HNC) [1–6]. In clinical practice, multimodal radiotherapies have been used to obtain an optimal dose distribution at the target, especially in the prostate, with satisfactory outcomes [1]. These therapies have been applied to HNC treatment [7, 8], but toxicities of the combined radiations to normal tissues are not fully known. Various methods [9–11] have been proposed to measure the dosimetry of a combined radiotherapy based on the assumption that the two therapies are delivered with suitable temporal separation to avoid synergistic tissue damages [9], but these methods have not been applied in HNC.

The management of HNC includes single-modality treatments for early stage tumors and multimodal therapies for advanced tumors. Such management must consider features that may adversely affect the prognosis, such as the depth of tumor invasion, tumor differentiation and the proximity of the tumor to the surgical margins [12–14]. According to the guidelines of the National Comprehensive Cancer Network [15], external beam radiotherapy (EBRT) for HNC is described as follows: definitive radiotherapy is 66–70 Gy for the primary tumor and 44–64 Gy for the neck via conventional fractionation (2.0 Gy/fraction, daily, Monday–Friday), and postoperative radiotherapy includes 60–66 Gy for primary and 44–66 Gy for the neck. The recurrence and persistence of HNC often lead to poor prognosis, because retreatment may be limited by the previous therapy, especially definitive radiotherapy, as the normal tissues might not tolerate well the repeated irradiation. For patients who have a residual tumor that cannot be completely resected, adjuvant treatment must be effective for local control. In addition to EBRT, a new modality should be proposed to address these needs.

The numbers of hypoxic cells and cells in the G_2_/M phase are critical for locoregional control. Fractional EBRT can kill sensitive cells effectively; however, these hypoxic cells with radiation resistance in tumors may pose a risk of recurrence, which results in failure of local control [16]. Interestingly, cell-killing effects induced by continuous LDR brachytherapy irradiation from ^125^I seeds, which mainly occur via apoptosis and G_2_/M cell-cycle arrest [17, 18], have been proposed. Moreover, the ^125^I interstitial LDR brachytherapy may arise as the most effective technique if it can increase the target dose while reducing the risk of complications in normal tissues [19]. Previous studies have reported that apoptosis and G2/M cell-cycle arrest induced by ^125^I seeds are the predominant mechanisms involved in the inhibition of tumor growth [20, 21]. In part, EBRT and LDR interstitial brachytherapy are complementary.

The combination of LDR brachytherapy and EBRT has been applied in the treatment of prostate cancer [1, 22, 23] and HNC [7, 8, 24]. However, radiation oncologists often find it difficult to use the combined forms as a feasible treatment of HNC, because normal tissue toxicities will be increased by the complex dose distribution [1]. Methods have been proposed to standardize the doses from EBRT and LDR brachytherapy based on the biologically effective dose or the equivalent uniform dose [9–11]. To minimize tissue toxicity, these methods could work by administering the therapies in an ordinal, not concomitant, manner. However, the synergistic effect caused by the multimodal radiotherapy could also be lost. If tissue toxicity is tolerable, physicians may prefer concomitantly combined radiotherapies for HNC treatment as it may approach a higher target dose with a promising prognosis. Therefore, it is important to study normal tissue tolerance to combined radiations of concomitant multimodal radiotherapies.

In the present study, the prescribed dose of ^125^I seed interstitial LDR permanent brachytherapy was maintained constant, whereas the added EBRT doses were administered at different levels to investigate the tolerance limit of normal neck tissues to combined radiations in a rabbit model.

## METHODS

### Instruments and reagents

^125^I seeds were obtained from Beijing ZhIBO Bio-Medical Technology Co., Ltd, China. These seeds had a radioactivity of 29.6 MBq (0.8 mCi) with a half-life of 59.4 days. The medical electronic accelerator was from Siemens Company, Erlangen, Germany (model name: PRIMUS). The Seed Implanting Brachytherapy System (SIBS) was provided by Beihang University, Beijing, China. The transmission electron microscope was from Hitachi, Tokyo, Japan (model name: HT7820). The anti-tumor necrosis factor alpha (TNF-α) antibody was from Abcam, USA (polyclonal antibody, Cat. No.: Ab199013). The diaminobenzidine histochemistry kit with streptavidin-horseradish peroxidase was from CW Bio-Medical Technology Co., Ltd, Beijing, China. The terminal deoxynucleotidyl transferase dUTP nick end labeling (TUNEL) Apoptosis Detection kit was from KeyGen BioTECH, Shanghai, China.

### Animals and implantation

Thirty-two Japanese male rabbits about 3 months old were purchased from Bio-Technology Co., Ltd, Wuhan, China. Laboratory animal care and the animal experimental procedures conformed to the ‘Chinese Administration Rules for Laboratory Animals’.

The rabbits were divided into two large groups, 20 as test and 12 as control. The animals in the test group received two treatments, ^125^I seed implants (four seeds per rabbit) first and EBRT in four dosages (50, 40, 30 and 20 Gy) with five animals per dose group (T_1_–T_4_) later. Pairs of seeds were contained in medical-grade silicone tubes and composed a chain. The seeds were surgically implanted into the neck of each rabbit and distributed as follows: one chain was placed by the internal jugular vein, and the other was fastened into the tracheoesophageal groove. Among the control animals, four as group C_1_ were implanted with just the ^125^I seeds without EBRT, four as group C_2_ were implanted with fake seeds in a similar manner and four untreated as group C_3_ constituted the blank control. Animals were anesthetized via ear vein injections of 3% pentobarbital sodium. The grouping method and data are presented in Table 1.

**Table 1:** Grouping and experimental data of the testing rabbits

Treatment Group	IOD for IHC (mean ± SD)	IOD for TUNEL (esophagus, mean ± SD)	Change in weight^a^ (kg) (mean ± SD)
50 Gy + ^125^I (5 rabbits, group T_1_)	0.0482 ± 0.0068	0.8499 ± 0.0777	0.75 ± 0.44
40 Gy + ^125^I (5 rabbits, group T_2_)	0.0419 ± 0.0074	0.7979 ± 0.0850	0.63 ± 0.21
30 Gy + ^125^I (5 rabbits, group T_3_)	0.0376 ± 0.0144	0.7745 ± 0.0796	1.12 ± 0.17
20 Gy + ^125^I (5 rabbits, group T_4_)	0.0353 ± 0.0183	0.7673 ± 0.0965	1.31 ± 0.34
^125^I (4 rabbits, group C_1_)	0.0304 ± 0.0226	0.7624 ± 0.0876	1.63 ± 0.22
Control (4 rabbits, group C_2_)	0.0133 ± 0.0086	0.4857 ± 0.0955	1.61 ± 0.10
Blank control (4 rabbits, group C_3_)	0.0134 ± 0.0054	0.4842 ± 0.0347	1.71 ± 0.19
Dead animals	0.0486 ± 0.0197	0.8108 ± 0.1618	
Statistical analysis	*F* = 2.812	*F* = 10.892	*F* = 11.378
	*P* = 0.041^*^	*P* < 0.001	*P* < 0.001

^*^ *P* < 0.05 is accepted as having a significant difference.

^a^weight_change_ = weight_Day84_–weight_Day0_

### Irradiation protocol

**Fig. 1:**
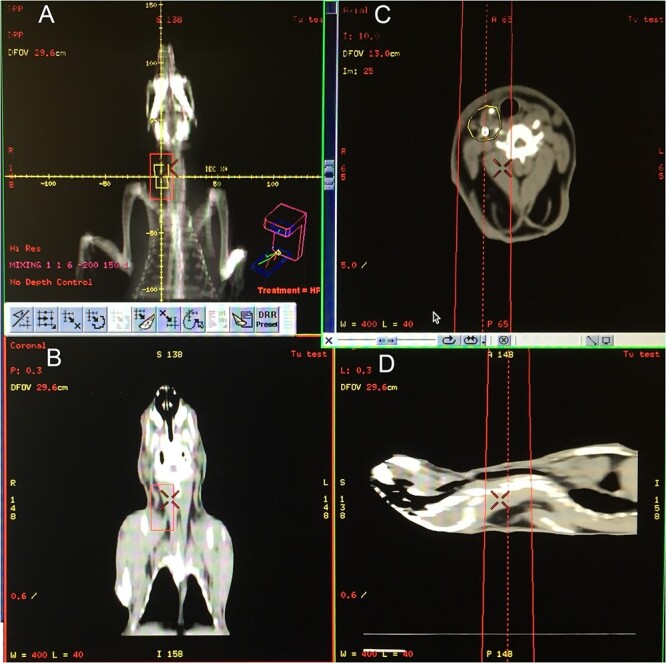
Rabbits were positioned on the CT simulator for radiotherapy simulation. (A) Radiotherapy plan created after CT scanning. The radiation field was determined to be 3 × 4 cm, with a 3 cm depth, and contained the trachea, esophagus, carotid artery, jugular vein, sternocleidomastoid muscle and other structures. (B–D) Display coronal, axial and sagittal CT, respectively, with the box line in neck showing the irradiation field. The high-density shadow in the yellow oval in (C) represents the seeds.

After the initial seed implantation, the experimental rabbits were treated with EBRT 3 weeks later. A computed tomography simulator was applied to locate the target. The target regions included the trachea, esophagus, carotid artery, jugular vein, sternocleidomastoid muscle and other structures (shown in Fig. 1). When administering the radiotherapy, the animals were under general anesthesia. A single dose of 2 Gy from a 10 MeV electron beam was applied via a single anterior field. The plan was administered as 2 Gy daily, 5 days per week.

### Radiation dose calculations

The SIBS was used to assess the quality of implantation. The Monte Carlo-aided dosimetry was adopted to measure the radiation dose using the ‘Dosimetry of Interstitial Brachytherapy Sources – AAPM Task Group No. 43 Report’ as a reference [25–27]. The initial dose, termed $\dot{\mathrm{D}}\,(\mathrm{r},\mathrm{\theta}$), was calculated according to the following equation:


$$ \dot{\mathrm{D}}\left(\mathrm{r},\mathrm{\theta} \right)={\mathrm{S}}_{\mathrm{K}}\wedge \left[\mathrm{G}\left(\mathrm{r},\mathrm{\theta} \right))/\mathrm{G}\left({\mathrm{r}}_0,{\mathrm{\theta}}_0\right)\right]\mathrm{g}\left(\mathrm{r}\right)\mathrm{F}\left(\mathrm{r},\mathrm{\theta} \right) $$

where S_k_ = 1.1, Λ is a constant parameter for ^125^I, Λ = 0.88 cGy·h^−1^·U^−1^, r denotes the distance between any point and the ^125^I particles, g(r) is the radial dose function and F(r,θ) is the anisotropy constant. Detailed data are not listed. After $\dot{\mathrm{D}}\left(\mathrm{r},\mathrm{\theta} \right)$ was confirmed for each seed, we summed the four different values of $\dot{\mathrm{D}}\left(\mathrm{r},\mathrm{\theta} \right)$ into $\dot{\mathrm{D}\ }$, and the dose delivered by the seeds was calculated with the following formula:


$$ \mathrm{D}=\frac{\dot{\mathrm{D}}\mathrm{t}}{\ln 2}\left(1-{2}^{\frac{-\mathrm{t}}{{\mathrm{t}}_{1/2}}}\right) $$

where D represents the total dose within time interval t. In our study, t equaled 90 days.

### Data collection and histopathological evaluation

We assessed the mortality and weight of all the rabbits at 1, 3, 5, 8, 10 and 12 weeks after implantation. All animals were euthanized via lethal injection 90 days after the operation. Tissues were removed from the target region, and a small section of the carotid artery was taken for electron microscopic examination and the rest of the samples were fixed in 10% neutral-buffered formalin, embedded in paraffin and cut into 4 μm thick slices for histopathological examination.

Immunohistochemistry (IHC) staining for TNF-α was performed according to the manufacturer’s instruction of the Streptavidin-HRP kit (DAB), and the primary antibody raised against TNF-α was diluted 1:20. The TUNEL assay was also performed to examine apoptosis using the Death Detection kit (KeyGen BioTECH) according to the manufacturer’s instruction.

Two expert pathologists examined all tissue sections. Degrees of staining were also measured based on the integral optical density (IOD) with computerized image processing by Image-Pro-Plus (6.0 version, Media Cybernetics, USA). The mean IOD of four high-power microscopic views (magnification ×400) was taken for each sample.

### Electron microscopy

Carotid artery samples were placed into 2.5% glutaraldehyde for 24 h, washed with phosphate-buffered saline (PBS, pH 7.4) and then fixed in 1% osmium tetroxide. After fixation, samples were washed with PBS and dehydrated in a series of increasing concentrations of acetone. Next, the samples were washed with propylene oxide and embedded in the epoxy resin embedding medium and solidified in an oven. Ultrathin sections were made with an ultramicrotome and were stained with uranyl acetate and lead nitrate, and then examined using the Hitachi transmission electron microscope.

### Statistical analysis

The distribution of data is expressed as the mean ± standard deviation (SD) or as a percentage. Differences between the groups were compared using a one-way analysis of variance (ANOVA) (SPSS version 22.0; SPSS, Inc., Chicago, USA), with *P* < 0.05 being considered significant. If the *P* value from the ANOVA was less than the significance level, a Tukey post hoc test [28, 29] was conducted to explore which groups were significantly different from each other. Survival curves were generated utilizing the Kaplan–Meier method and compared using the log-rank test. All *P* values <0.05 were considered statistically significant.

## RESULTS

### Calculation for dosimetry

**Fig. 2:**
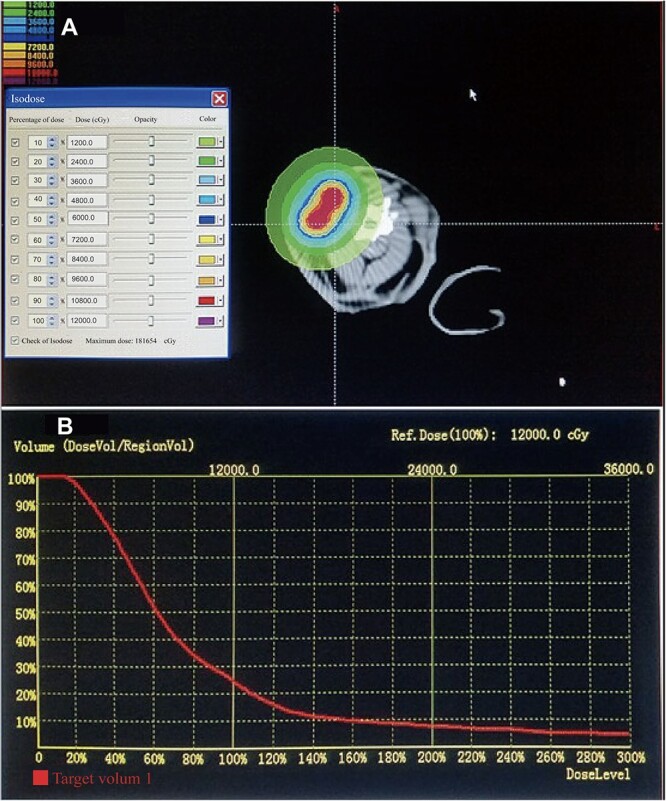
(**A**) A typical isodose curve of four seeds. (**B**) Dose-volume histograms.

Animals in groups T_1_–T_4_ and C_1_ were successfully implanted with the seeds. The SIBS was used to assess the quality of implantation by forming dose-volume histograms and to verify the isodose curves. An imaginary tumor target was delineated (with a width of 3 cm) by setting the seeds as the center. The minimum peripheral dose (mPD) was 17.6 Gy, and the maximum dose was 1812.5 Gy (which occurred very close to the seeds, such as at the carotid artery and to the right of the trachea), and the D90 was 34.5 Gy. The mean dose for the target region was 124.5 Gy (see Fig. 2). We calculated doses at the target area that contained the samples using the method described in the Methods section. The minimum total dose was 195.3 Gy in 90 days, and most of the area had a considerably higher dose, over 250 Gy, which greatly exceeded doses prescribed for normal tissues in clinical practice.

### Mortality and weight changes

**Fig. 3:**
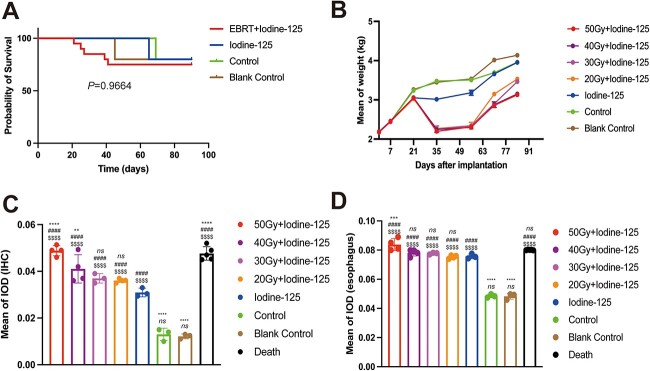
(**A**) The survival curve. All groups showed no significant difference in survival rate by log-rank test (*P* = 0.9664). (**B**) Changes in mean weight (kg). After EBRT was administered, mean weights of the rabbits in groups T_1–4_ decreased sharply, and two weeks later, increased gradually, suggesting that they might have recovered from or adapted to the damages caused by radiotherapy. However, the mean weights of the rabbits in groups C_1–3_ increased continuously. (**C**) Compared with other groups, the C_2_ and C_3_ control groups exhibited significantly lower IODs in the IHC analysis. (**D**) Groups C_2_ and C_3_ had significantly lower IODs in the TUNEL analysis than other groups. The labels over the box ^*^, # and $ indicate the difference of each group compared with C_1_, C_2_ and C_3,_ respectively. ^*^^*^^*^^*^, ####, $$$$*P* < 0.0001. ^*^^*^^*^P < 0.001. ^*^^*^P < 0.01. ns: no significance.

During the EBRT treatment, five rabbits died in the four test groups (one per group, except that two died in group T_3_), and three rabbits died in the three control groups (one per group), with about equal mortality in all groups with no significant differences between each other (*P* = 0.9664, Fig. 3A). In surviving rabbits, none displayed apparent pathological abnormality. Among the five dead animals of the test group, four deaths occurred when a dose of 10 Gy was reached (1 rabbit each in groups T_1_–_4_), and the other death occurred at 6 Gy (in group T_3_). All deaths occurred before the completion of the prescribed dose. The mean weight of the rabbits in groups T_1–4_ decreased significantly after the EBRT treatments were administered, but increased gradually 2 weeks later. However, the mean weights of the rabbits in the three control groups increased continuously. In addition, there was a significant difference in weight change between T_1–2_ and T_4_, C_1_, C_2_, C_3_ (Fig. 3B, Supplementary Fig. 2).

### Histopathological assessment

**Fig. 4:**
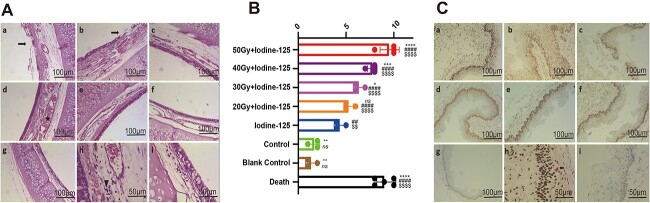
HE staining and TUNEL assay. Panels a–g represent groups T_1–4_ and C_1–3_, respectively (100×, 100 μm); panels h and i show the higher magnification for group T_1_ and the blank group C_3_, respectively (200×, 50 μm). (**A**) HE stain of the trachea. Pathological changes, such as epithelium exfoliation (arrows in images a and b), blood vessel expansion (star in image d), the gathering of plasma cells (arrowhead in image h) and a reduction in goblet cells are seen. (**B**) Score of tracheal pathological damage (HE staining). The C_2_ and C_3_ control groups exhibited significantly lower score than other groups in tracheal pathological damage. The labels in the right of box ^*^, # and $ indicate the difference of each group compared with C_1_, C_2_ and C_3_. ^*^^*^^*^^*^, ####, $$$$*P* < 0.0001. ^*^^*^^*^P < 0.001. ^*^^*^, ## and $$*P* < 0.01. ^*^P < 0.05. ns: no significance. (**C**) TUNEL of the esophageal mucosa. The region of brown-yellow granules presents in the esophageal mucosa layer exhibited a decreasing trend from groups T_1–4_ and C_1_. Apoptosis also occurred in the control groups C_2_ and C_3_ (see images f and g), frequently in the basal layer region, suggesting that apoptosis is related to the rate of cell proliferation when irradiation is administered.

Visual examination did not discover necrosis, perforation or edema in any of the rabbits. Hematoxylin and eosin (HE) staining of tissue sections showed that the esophageal mucosa in groups T and C_1_ was thickened, and more new vessels appeared in comparison with the control groups of C_2_ and C_3_. These phenomena tended to increase with the radio dose absorbed by the tissue. In the trachea, the mucosal epithelium was exfoliated, goblet cells were less abundant, blood vessels were expanded and congested in the submucosa. In addition, swelling of the tracheal mucosal and mucosal edema were observed. These findings were related to the relative position of the seeds and the EBRT dose. Fewer white blood cells were observed in the submucosa (Fig. 4A). Based on the pathological changes of the trachea, we scored these pathological changes and developed a quantitative standard to evaluate the tissue damage quantitatively (Supplementary Material, Table S1). The score of tracheal pathological damage in groups C_2_ and C_3_ was significantly lower than the other groups, and except for the T_4_ group, the pathological score of the T groups was significantly higher than that of the C_1_ group. Among T group, T_1_ had a higher score than others. T_2_ showed a higher score compared with T_4_, but the difference was not significant between T_2_ and T_3_, T_3_ and T_4_ (*P* < 0.05, Fig. 4B, Supplementary Fig. 2). Pathological changes in the carotid artery, jugular vein, vagus nerve, gristle and muscle tissue were not obvious under an optical microscope. Furthermore, in the rabbits that accidentally died, pathological changes did not appear to be different from those in the surviving rabbits.

### IHC staining and TUNEL assays

IHC staining assessed inflammatory reactions. The IHC staining revealed brown-yellow TNF-α-positive particles located predominantly in the esophageal and tracheal mucosa, especially the staining of esophageal mucosa being more obvious (Supplementary Fig. 1). However, the staining of cartilage, muscle, connective tissue, esophageal muscular layer, jugular artery and vein was difficult to identify. Mean IOD values of esophageal mucosa are listed in Table 1. TNF-α expressions in groups T and C_1_ were significantly increased compared with those in groups C_2_ and C_3._ In addition, T_1–2_ groups had a higher expression of TNF-α compared with C_1_, whereas T_3–4_ showed no significant difference compared with C_1_. In T group, T_1_ had a higher expression of TNF-α than other T groups, and T_2_, T_3_, T_4_ showed no significant difference according to multiple comparisons (Fig. 3C, Supplementary Fig. 2).

The TUNEL assay detected apoptosis, which occurred primarily in the esophageal mucosa (Fig. 4C). In groups T and C_1_, the range of apoptotic cell distributions in the esophageal mucosa corresponded to the dose of radiotherapy, and there were significant differences between the groups, especially, C_2_ and C_3_ had lower apoptotic rate than other groups. However, when compared with the C_1_ group, the T groups showed no difference in TUNEL assay. T_1_ had a higher apoptotic rate than the other T groups, and there was no significant difference among other T groups according to multiple comparisons (Fig. 3D, Supplementary Fig. 2). In the trachea, a dispersed distribution of apoptotic cells was observed. Apoptotic cells were rarely observed in the vessels, mesenchyme and muscle tissue.

### Electron microscopy

**Fig. 5:**
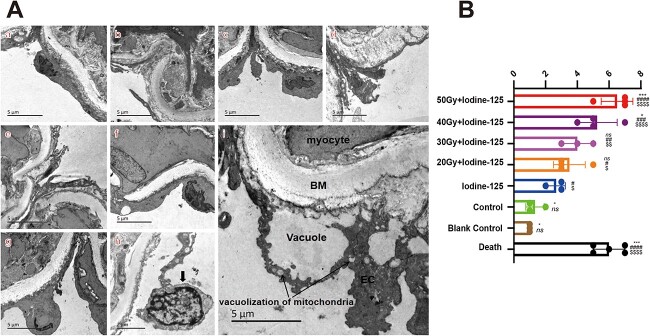
Electron microscopy of carotid arteries. (**A**) Panels a–g show the electron microscopic appearance of groups T_1–4_ and C_1–3_, respectively (5000×). ECs became swollen and were shed from the BM, and vacuolization occurred in the mitochondria and the tunica intima (T_1_ group, arrow showed in i, 5000×). Nuclear chromatin concentration and edge accumulation (T_1_ group, chromatin margination) were observed (downward arrow in h, 10 000×). (**B**) Score of damage of carotid arteries. Groups C_2_ and C_3_ had significantly lower score in the analysis of carotid arteries’ damage than other groups. The labels in the right of box indicate^*^, # and $ the difference of each group compared with C_1_, C_2_ and C_3_, respectively. ####, $$$$*P* < 0.0001. ^*^^*^^*^P < 0.001. ^*^^*^, ## and $$*P* < 0.01. ^*^, # and $*P* < 0.05. ns: no significance.

Electron microscopy was performed for carotid arteries. Endothelial cells (ECs) in groups T_1–4_ and C_1_ were not flat; they became swollen, and some exfoliated from the basement membrane (BM). Cellular organelles appeared injured, including vacuolization in the mitochondria and an extension of the rough endoplasmic reticulum. Although there was fibrin deposition in the mucosal layer and BM incrassation (edema), myocytes were not impaired (Fig. 5A). As for the HE staining of tracheal pathological damage, we also developed a scoring protocol. By performing this scoring system, we found that the scores of carotid artery pathological damage in groups C_2_ and C_3_ were significantly lower than those in other groups. In addition, the T_1–2_ groups had a higher score when compared with C_1_, but the difference was not significant between T_3–4_ and C_1_. T_1_ displayed a higher score than T_3_ and T_4_; however, T_2_, T_3_ and T_4_ had no significant difference when compared with each other (Fig. 5B, Supplementary Fig. 2). No other tissue damages were noticed.

## DISCUSSION

In this study, premature animal death occurred randomly in all groups. Before death occurred, these sick rabbits exhibited loss of appetite and body weight, apparent hypothermia and reduced activity for 1–3 days. During the postmortem examination, we did not find any direct lethiferous impairments. We speculate that multiple factors could have caused these premature animal deaths, including: (i) systemic reactions to the experimental therapy; (ii) toxicity accumulation of pentobarbital sodium; (iii) loss of appetite caused malnutrition; and (iv) environmental factors such as humidity and temperature changes. Because these mortalities did not significantly differ between groups, the random animal deaths should not affect the result of this study.

The HE staining revealed that the degree of pathological damage was almost consistent with the total dose absorbed by normal tissues. White blood cells in the acute inflammation phase were not noticeable at the treatment endpoint, but some plasma cells could be seen, suggesting a later stage of inflammation or the presence of chronic inflammation. Many published papers have reported that TNF-α, an inflammatory cytokine, increases in response to radiotherapy [30, 31]. Bao *et al*. [32] reported that, in lung tissues, the intensity of TNF-α expression increased from 2 h to 4 weeks after a 2-Gy radiation dose. We observed strong positive staining for TNF-α in the esophagus and trachea in groups T_1_ and T_2_. Although there was no statistical difference between the T_3–4_ group and the C_1_ group, we could still see a trend that the expression of TNF-α gradually decreased with a reduction in radiation dose. Staining was also not noted in groups C_2_ and C_3_, suggesting that the inflammation is related to the dosage of radiotherapy. The results of HE staining and IHC suggested that the degree of pathological damage and inflammatory reaction of normal tissue in the rabbit neck caused by radiotherapy may be related to the dose absorbed by the tissue.

DNA is the target of radiation. The cell death induced by radiation appears to occur via various mechanisms. Apoptosis may be a major mechanism by which radiotherapy works, especially for continuous LDR irradiation [33]. Paris *et al*. [34] reported that endothelial apoptosis is the primary lesion that initiates intestinal radiation damage in mice. Ma *et al*. [35] demonstrated that ^125^I induces apoptosis by regulating p53, microvessel density and vascular endothelial growth factor levels in colorectal cancer. In an *in vitro* trial, Wang *et al*. [18] reported that the radiobiological effects induced by ^125^I seeds are most likely due to apoptosis and G2/M cell-cycle arrest. In addition to brachytherapy, the present study included additional EBRT in the test groups. Theoretically, this combined therapy should produce overlapping effects on normal tissues in rabbits. Consistently, we found higher rates of apoptosis in esophageal and tracheal mucosae in test animals. However, the mucosae of animals of group C_1_ were also hyperchromatic, but those of groups C_2_ and C_3_ were lightly stained. Fewer stained particles in the other areas of normal tissue were observed, suggesting that apoptosis occurred in tissues with a faster turnover rate.

Early responding tissues containing rapidly dividing cells are highly sensitive to radiation, whereas late responding tissues with slower turnover rates are less sensitive. In this study, pathological injuries to carotid arteries mainly occurred in the endodermis, and myocyte changes were seldom seen. Due to limitation of time required for evaluating long-term toxicity to normal tissues, we did not study late-stage radioactive complications. Late-stage radioactive complications include tissue fibrosis, arterial rupture and necrosis. In clinical practice, the LDR permanent brachytherapy is performed to combine with or follow EBRT. Based on the theory of biological effects (converting the prescription dose into biological effects), the LDR permanent brachytherapy can be regarded as or equated with re-irradiation administrated by EBRT. Some reports [36, 37] showed that, in patients with recurrent HNC who accepted re-irradiation by EBRT, the median prescription dose of initial radiation is 66–68.4Gy, and the median re-irradiation dose of EBRT can reach 60Gy, and treatment-related fatalities (such as carotid artery blowout) were rare. However, we still need to pay enough attention to these fatal late toxicities of radiation therapy. Vissink *et al*. [38] reported that normal cells have a greater capacity than tumor cells to repair radiation damage, especially at low doses. In addition, the mechanisms underlying the biological effects caused by LDR permanent brachytherapy are likely distinct from those of EBRT, which works in a ‘moderate’ manner that provides a chance to repair sublethal damage in a timely manner. Therefore, a comparatively low-dose EBRT combined with LDR permanent brachytherapy should be feasible in clinic practice.

In this study, the highest EBRT dose was 50 Gy, and the doses of LDR permanent brachytherapy were higher than 195 Gy. Both doses in this combined radiotherapy were higher than those reported previously [7, 8]. Although these data are based on animal studies, and due to some technical reasons, we failed to design a rabbit model with transplanted tumors. Nevertheless, we suggest that a limited dose of EBRT combined with LDR is feasible in patients with HNC, provided that patients are closely observed for possible complications and that any complications are promptly treated. Therefore, we propose that a combined radiotherapy between the LDR brachytherapy and EBRT is appropriate for the following cases:

(i) patients with advanced HNC exhibit residual tumors requiring a salvage surgery and a high-dose radiation therapy.
(ii) patients with a malignancy that extensively invades the adjacent tissues, including the great vessels.
(iii) patients who have persistent and recurrent HNC after non-definitive radiation therapy are treated with surgical resection; however, they still require additional radiotherapy and brachytherapy for treating local regions combined with a low dose of EBRT for the neck.
(iv) patients who have persistent and recurrent HNC after definitive radiation therapy with or without surgical resection, regardless of residual tumor, can still receive an interstitial ^125^I seed implantation alone as brachytherapy.

Our original intention in developing this animal model was to administer a high-dose radiation to the primary lesion via brachytherapy and to use constrained intensity-modulated radiation therapy for the uninvolved areas. We hope that this method would provide patients with a promising prognosis.

## CONCLUSION

Because the normal head and neck tissue of the rabbit tolerated well a maximal dose of 50 Gy of EBRT combined with therapeutic LDR permanent brachytherapy, the combined radiotherapy appears to be safe in the animal model. Therefore, in human patients, the combination between the LDR permanent brachytherapy and limited EBRT may be a feasible therapeutic option.

## Supplementary Material

Supplementary_Figure_1_rrad036

Supplementary_figure_2_rrad036

Supplementary_table_S1_rrad036

## Data Availability

The data of this study can be obtained from the author upon reasonable request.
