# Supplementary figures and images for: Tolerance limit of external beam radiotherapy combined with low-dose rate brachytherapy in normal rabbit tissue
Source: J Radiat Res. 2023 May 20;64(4):651–60. doi: 10.1093/jrr/rrad036 (PMC10354847; doi:10.1093/jrr/rrad036)

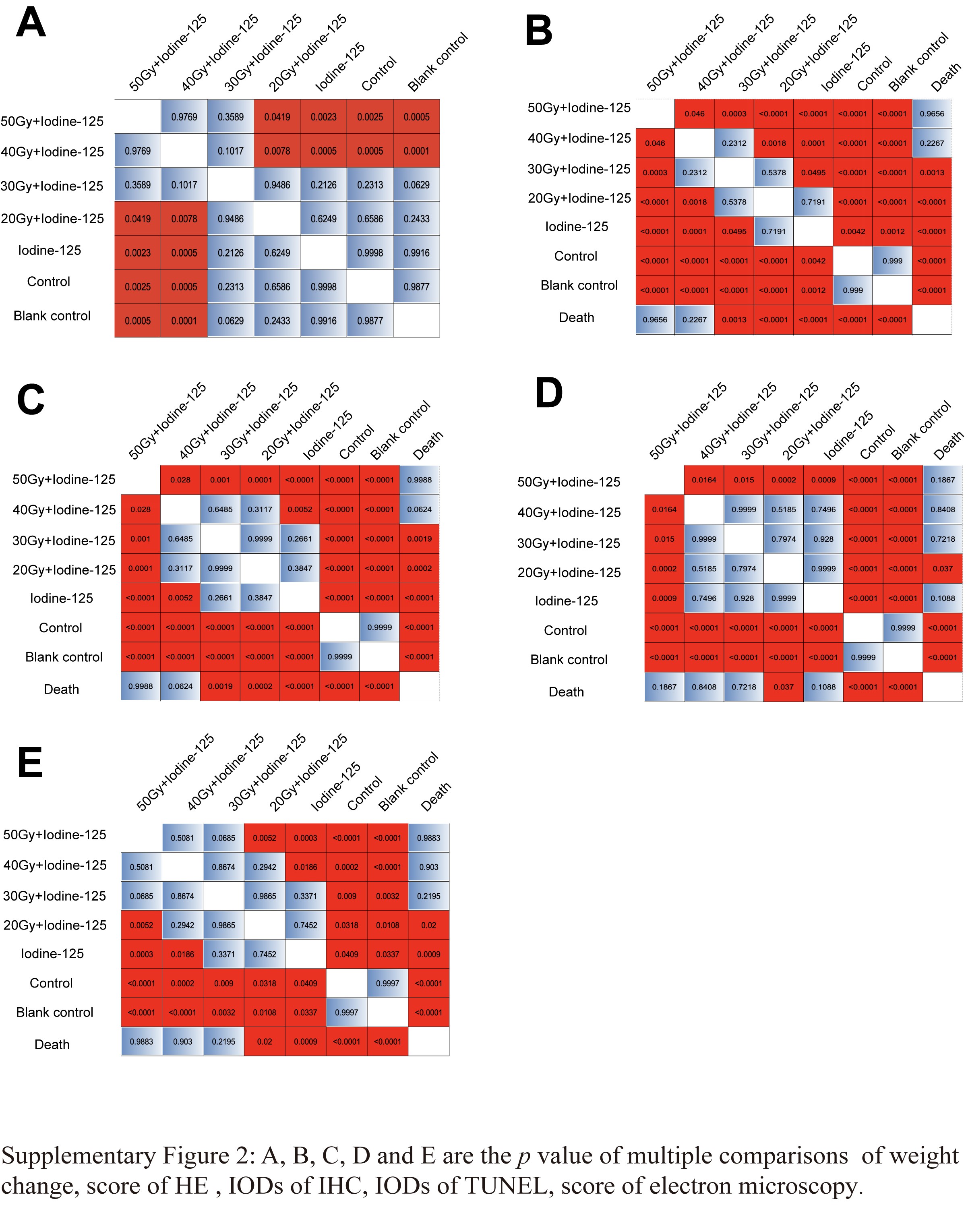

Supplement: Supplementary_figure_2_rrad036 [file supplementary_figure_2_rrad036.jpeg]
