# Supplementary material for: Tolerance limit of external beam radiotherapy combined with low-dose rate brachytherapy in normal rabbit tissue
Source: J Radiat Res. 2023 May 20;64(4):651–60. doi: 10.1093/jrr/rrad036 (PMC10354847; doi:10.1093/jrr/rrad036)
Supplement: Supplementary_table_S1_rrad036 [file supplementary_table_s1_rrad036.docx]

**Scoring rules for tracheal damage（HE staining）and** **damage of carotid arteries（Electron microscopy）**

| **HE staining** | | | | **Electron microscopy** | | | |
| --- | --- | --- | --- | --- | --- | --- | --- |
| Damage | Degree of damage | Score |  | Damage | Degree of damage | Score |  |
| Exfoliation of tracheal mucosa* | Sequential large fragments of mucosal exfoliation | 4 |  | Endothelial cell shedding* | Yes | 2 |  |
|  | Patchy or punctate mucosal exfoliation | 2 |  |  | NO | 0 |  |
|  | No mucosal exfoliation | 0 |  |  |  |  |  |
| Submucosal vasodilation and hyperemia | Vasodilation and accumulation of blood cells | 2 |  | Nuclear chromatin concentration and edge accumulation* | Yes | 2 |  |
|  | Only mild vasodilation | 1 |  |  | NO | 0 |  |
|  | No vasodilation | 0 |  |  |  |  |  |
| Swelling of the tracheal mucosa | Yes | 1 |  | Endothelial cell swelling | Yes | 1 |  |
|  | NO | 0 |  |  | NO | 0 |  |
| Decreased goblet cells | Yes | 1 |  | Mitochondrial vacuolation | Yes | 1 |  |
|  | NO | 0 |  |  | NO | 0 |  |
| Mucous membrane swelling | Yes | 1 |  | Basement membrane incrassation | Yes | 1 |  |
|  | NO | 0 |  |  | NO | 0 |  |
| Leukocyte infiltration | abundant | 1 |  |  |  |  |  |
|  | rare | 0 |  |  |  |  |  |

*** represents severe injury, which will calculate a high score.**
